# Interpreting machine for ambiguous figures

**DOI:** 10.1016/j.heliyon.2023.e16949

**Published:** 2023-06-07

**Authors:** Yehuda Roth

**Affiliations:** Oranim College, K. Tivon 36006, Israel

## Abstract

Quantum theory presents a unique scenario pertaining to collapse processes. A device that measures variables incompatible with those being detected collapses randomly into one of the states defined by the measuring device. The distinction that a collapsed output is not an accurate description of reality but rather a random selection from a set of values derived from the measuring device allows us to utilize the collapse process to propose a scheme wherein a machine becomes capable of performing interpreting processes. We present herein a basic schematic of a machine that demonstrates the principle of interpretation relying on the polarization phenomenon of photons. The operation of the device is demonstrated using an ambiguous figure. We believe that building an interpreting device can contribute to the field of AI.

## 1 Introduction

A quantum-like model is presented to describe a cognition and decision-making model. The term “quantum-like” is used to distinguish this model from actual physical processes [1]. In this manuscript, we suggest the implementation of these procedures to plan a quantum-based interpreting machine.

According to the principle of objectivity in science, behavioral sciences assume that results discovered in controlled conditions also apply outside the laboratory (uniformity assumption) [2]. The term “interpretation” refers to what is known as “private events” and to behavioral phenomena observable only to the behaving organism. As the agreement between observers of these events according to traditional science is impossible, they pose a challenge to experimental science [2], [3].

Interpretation plays a crucial role in several aspects of life [4]. The interpretation of reality has a decisive role in an individual's understanding of reality [4]. Therefore, a considerable part of the communication between social people relies on their personal interpretations [4]. Thus, essential aspects of human communication rely on individual interpretations.

The traditional approach to intelligence is perceived as an abstract ability of the individual's mind based on a mechanism for rational thinking. In other words, the same data fed into a rational process will produce the same result in contrast to the interpretation process described above. After realizing that most processes affecting human communication are interpretation-based, a significant part of the ideas concerning current artificial intelligence research is currently prioritizing social relationships as a critical and constructive component of intelligent behavior. This concept requires the consideration of the implementation of a machine capable of interpreting reality [5]. Based on the quantum collapse scenario [6], [7], [8], we propose a formalism to describe the process of interpretation. Accordingly, we propose a quantum-based interpreting machine to implement this formalism.

We present a formalism describing the interpretation process based on quantum principles. We propose the application of quantum-like formalism [1] to build a quantum-based machine capable of performing personal-like interpretations, where the term “persona-like” refers to the machine which performs the interpretation process instead of a human. Additionally, we demonstrate that this individuality-like behavior is due to quantum measurements [6], [7], [8] performed by the interpreting machine. The main result of our formalism is that the only one who reads the collapse output is the observer defined by the interpreting machine (similar to the definition provided for the term privet event [2], [3]).

**Figure 1:**
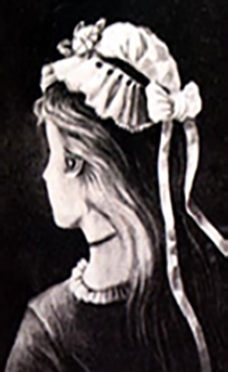
Example of an ambiguous figure: Is it Olive or yana?

**Figure 2:**
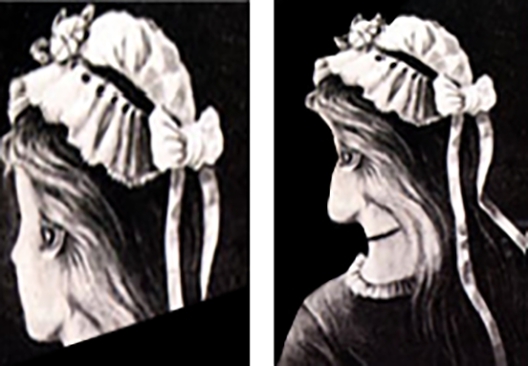
Left: Image of a young lady (Yana). Right: Image of an old lady (Olive).

We demonstrate the findings of our study using ambiguous figures, as shown in Fig. 1. These ambiguous figures cause a visual interpretation between several separate image forms [9], [10], [11]. We focus on the ambiguous figure of “my wife and my mother-in-law” shown in Fig. 1 [12], [13]. In a preliminary observation of Fig. 2, we recognize a young (named Yana) and an old lady (named Olive). However, Fig. 1 shows that our perception reverses spontaneously in the cases of the two lady images [9]. Thus, similar to the quantum-collapse scenario in which the measurement's output collapses into a single option, we associate this “Yana–Olive” spontaneously reversed perception with the quantum-like collapse defined above. Finally, we propose a sketch of a machine capable of interpreting vague images. That is, we determine whether the ambiguous Fig. 1 describes Olive or yana.

## 2 Interpretation activities

The perception process occurs in three phases: selection, organization, and interpretation [14], [15]. In this study, we refer to all these activities as an expression of interpretation. We selected slightly different phases, namely, state construction, classified representation, representation, and determination based on quantum formalism. We demonstrate these definitions using the Olive–yana shown in Fig. 1 [9], [10], [11], [16], [17] where according to the Merriam—Webster dictionary, an ambiguous figure is a picture of a subject that the viewer may see and perceive to comprise either two different issues, or the same subject from two different viewpoints depending on how the total configuration is interpreted [18]. The adapted-quantum-like stages of the interpretation processes are:

1. **State Construction**
  In state construction, the system transforms the items to be interpreted into a state in a Hilbert space. This state is denoted as . In this study, details describing a transformation from an object (such as a simple image) into a state are not discussed. However, a procedure for generating coherence was described in [19], where a nonlinear approach was implemented.
  In our demonstration:
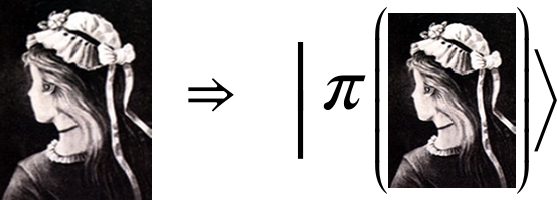
2. **Classified representation** The system defines the states (concepts) to be used to interpret the information received.
  The concepts are defined by the states $|I_{i}\rangle$ where *i* labels the state in the corresponding Hilbert space. In our demonstration, the classified concepts generated a space describing women age with the corresponding states, |o〉 and |y〉.
  In our demonstration,
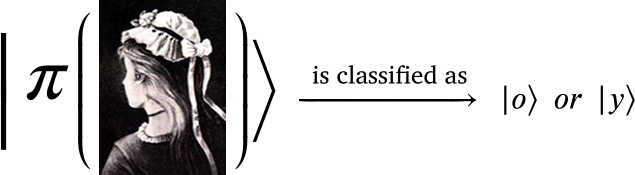
 where the states |o〉 and |y〉 denote the old and young women, respectively.
3. **Representation**
  The constructed state is represented in terms of classification states. Defining
  (1) $$R\overset{\text{def}}{=}\sum_{i}|I_{i}\rangle \langle I_{i}|$$
  where $|I_{i}\rangle$ describes the classified states with whom the observer describes the measured item. As a classification operator, we obtain,(2)
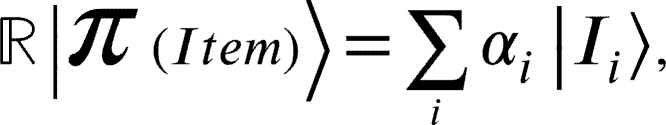
 with,(3)
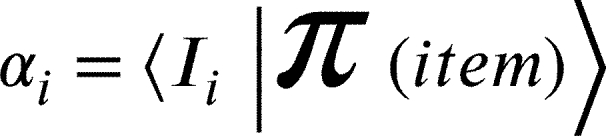
 In the demonstration, we consider $|I_{o}\rangle =|o\rangle$ and $|I_{y}\rangle =|y\rangle$ to obtain,(4)
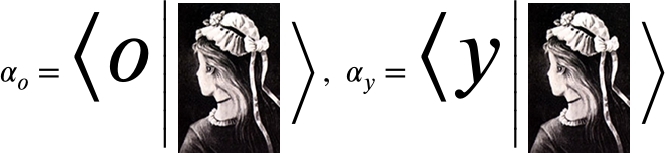
 where according to quantum formalism, ${\left|\alpha_{o}\right|}^{2}$ and ${\left|\alpha_{y}\right|}^{2}$ are the probabilities for  to be interpreted as a young or an older woman, respectively.
4. **Determination**
  The state collapses into one of the classification states to complete the interpretation.
  We use the observable
  (5) $$D\;=\sum_{i}\iota_{i}|I_{i}\rangle \langle I_{i}|,$$
  where $\iota_{i}$ are *eigenconcepts* that serve as the measurement output (similar to [20]). In the determination process, out of *N* alternatives, only one value is obtained; we will mark this with the letter *k*. Accordingly, we have(6)
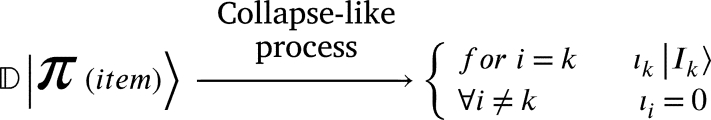
 These are the interpretation results provided to the observer.
  In our demonstration,(7)
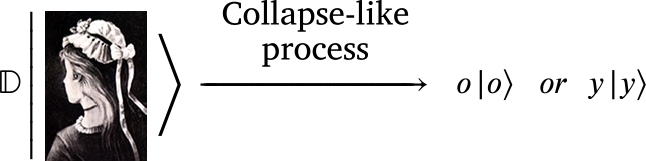
 where *o* and *y* are the eigenvalues. Visually, D operates such that,(8)
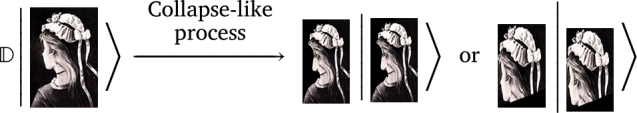
 where now  and  are the eigenconcepts.

## 3 Interpreting machine scheme

In this section, we propose a scheme for a machine that interprets the ambiguous figure of Fig. 1 into a single output.

### 3.1 Orthogonality in Yana and Olive images

In our perception, a lady can be either young or old; the two situations cannot exist simultaneously. From an algebraic perspective, this is the definition of orthogonality responsible for the ambiguity. However, in the separate images of Olive and Yana, using the definitions of the pixel states, we recognize no orthogonality. Moreover, because there are numerous shades overlapping equivalent pixels in both images, we realize that the two images are far from orthogonal. Orthogonality appears only at the interpretation stage, where the pixel collection of Figure 2, Figure 1 are classified in the concept “ladies age.” These concepts are orthogonal because a lady cannot be old and young simultaneously. Displaying the images only after they are classified as the states |o〉 for Olive and |y〉 for Yana, they are orthogonal, i.e., 〈o|y〉=0. By defining the states |π(Image)〉 as image states presented in the pixels' representation, we obtain(9)
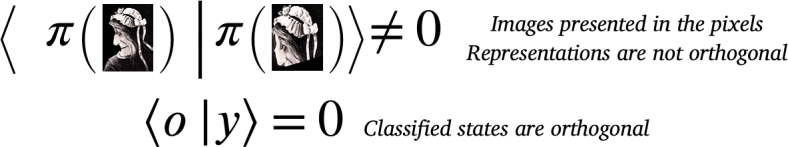


### 3.2 Pixel basis of states

We recall that in the state-construction stage, the received data are represented by a state in the Hilbert space. The purpose of this section is to demonstrate the feasibility of translating an image (as on paper) into a state belonging to a Hilbert space.

Our description consists of two spaces: a 1*D Hue-space* representing shade intensities where states in one space can be projected onto the second space composed of a spanning set referred as to *a pixel basis of states.*

To clarify this concept, the definition of pixels and hue states is unrelated to the physical nature of electromagnetic radiation. It is simply a mathematical representation of images printed on paper or displayed on a screen. For simplicity, we present only a mathematical model describing black-and-white images.

The requirements for orthogonal states necessitate negative amplitudes. To facilitate this, we set a zero-valued amplitude in the hue space to be gray so that darker and lighter states (compared with the background stage) have positive and negative amplitudes, respectively. The Hilbert space that we associate with the hue-space consists of the single state |η〉. The intensity of the hue is determined by a state amplitude; for consistency with the Hilbert space, a state amplitude such as that of the state A|η〉 is chosen, and the hue intensity is determined by the factor $A^{2}$ (we find no reason to define complex amplitudes). To demonstrate the relation between |η〉 to hues, consider *A* as an amplitude such that |ψ〉=A|η〉. We then have,

To define the pixel basis of states, we divide the image into squares (pixels). Each pixel is associated with a state |i,j〉 with i,j identifying a pixel position in an image matrix. By implementing the unity operator $\sum_{i,j}|i,j\rangle \langle i,j|$ over |η〉, we obtain the image state |ϕ〉:

(10) $$|\phi \rangle =\sum_{i,j}P_{i,j}|i,j\rangle ,\;\;\;\;\;P_{i,j}=\langle i,j|\eta \rangle$$

According to our definition, the strength of a shade in the pixel i,j is determined by the corresponding factor $P_{i,j}^{2}$.

## 4 Description of an interpreting device

**Figure 3:**
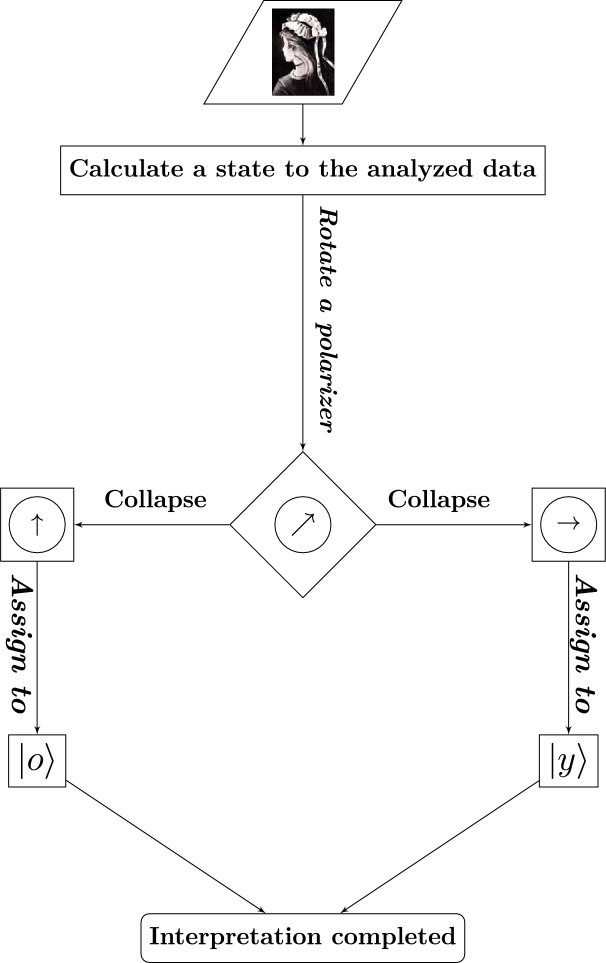
Flowchart describing the interpreting device. The assignments to |*y*〉 or |*o*〉 were selected arbitrarily.

Thus far, we have introduced the theoretical background describing the interpretation process. Its implementation is now demonstrated using a simple optical device capable of interpreting ambiguous images, as described in the flowchart in Fig. 3.

The device consists of a computational unit and two polarizers. The computational unit receives light from the image and analyzes it by dividing it into pixels such that a pixel's set characterizes each image. Each of those is associated with an angle, and then the first polarizer is rotated to this specific angle to form a state out of the light passing through. A second polarizer with a predefined direction is placed behind the adjustable polarizer. It represents the modes of translation of the figure. For example, horizontal and vertical polarization denote a young and mature woman, respectively. A photon that passes through the entire process eventually emerges with vertical or horizontal polarization, that is, as either a young or mature woman. In the following subsections, we follow the interpretation stages as presented above.

### 4.1 State construction

In an initial, digital imaging process, small squares (pixels) are assigned to the figure to be interpreted. Measuring the light intensity $I_{\pi }\;\left(\pi =(i,j)\right)$ arriving from each pixel, an amplitude $P_{\pi }$ is calculated as described below. Given the intensity range $I_{\pi }\pm \Delta I$, where ΔI is the device resolution, the amplitudes are calculated as $P_{\pi }=\pm \sqrt{I_{\pi }}$ where *P* respectively has negative or positive signs above or below the zero-state defined by the gray hue (see Table 1).

**Table 1:** Examples of hue states.

Amplitude (*A*)	Hue (*η*)
1	

0	

−1	

At the end of this process, the computer memory possesses a set of amplitudes $\left\{\phi \right\}=\left(P_{1}^{\left(\phi \right)},P_{2}^{\left(\phi \right)},...P_{\pi }^{\left(\phi \right)},...\right)$ that is identified with an image denoted as *ϕ*. To translate the set {ϕ} into a quantum state, the device rotates an adjustable polarizer at an angle $\theta_{\phi }$ defined in advance by a predefined rule that is stored in the device. This rotated polarizer associates all passing photons with a quantum state $|\theta_{\phi }\rangle$ to complete the stage of state's construction.

In our demonstration:
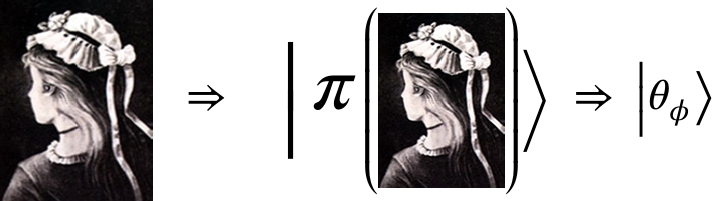


### 4.2 Representation and determination

To define the positions to which the image will be translated, there is an additional polarizer in the device, which unlike the adjustable polarizer, is fixed in orientation. This polarizer defines the states into which the image will be interpreted.

If we assume, for example, that this polarizer transmits photons only with polarization in the horizontal orientation, we can calibrate it to show the states into which the image will be interpreted such that photons passing through it can be associated with the state |o〉, and the absence of photons on the vertical axis can be associated with |y〉.

A photon that passes through the rotated polarizer will be in a state $|\theta_{\phi }\rangle$. Traveling through the second polarizer will cause it to collapse into one of the device states to determine the interpretation outcome.

### 4.3 Hilbert space of the constructed states

In quantum computers, unitary and hermit operators play the role in the implementation of observable and logical gates [21]. To implement our system, we derive herein an operator form describing the interpretation process.

Suppose we have two sets $\left\{\phi_{1}\right\}$ and $\left\{\phi_{2}\right\}$. Considering the relationship $\left\{\phi_{3}\right\}=A_{1}\left\{\phi_{1}\right\}+A_{2}\left\{\phi_{2}\right\}$ where *A*'s are arbitrary amplitudes, we notice that $\left\{\phi_{3}\right\}$ can also be used as a set in constructing the device state. Thus, we can define a superposition between sets and also consider all the sets as a closed system for all superposition sets. Implementing the Dirac notation for {ϕ} we can define the inner product as

(11) $$\langle \phi_{1}|\phi_{2}\rangle =\sum_{i}P_{i}^{\left(\phi_{1}\right)}P_{i}^{\left(\phi_{2}\right)}.$$

To complete the Hilbert-space definition, we consider the minimum number of sets that compose all the possible sets as the space's spanning set.

Consider |θ〉 as the space describing all possible polarizer rotations. Considering that |θ〉 and |ϕ〉 respectively denote the parts of different spaces Θ and Φ, we generated the outer product space Φ⊗Θ spanned by the states:

(12) |ψ〉=|θ〉|ϕ〉

Thus, we represent the angle assignment with the operator

(13) $$\Theta =\sum_{\phi }\theta_{\phi }|\phi \rangle \langle \phi |$$

to obtain,

(14) $$\Theta |\psi \rangle =\theta_{\phi }|\psi \rangle$$

We refer to **Θ** as the *executive component* operator where the operator's input is the calculated state |ϕ〉 with the eigenvalue $\theta_{\phi }$ being the rotational angle.

An adjustable device—a *controller* component assigns the planned rotational angle *θ* with the *ϕ* input. This is represented by the following operator

(15) $$\Phi =\sum_{\theta }\phi_{\theta }|\theta \rangle \langle \theta |$$

to obtain,

(16) $$\Phi |\phi \rangle =\phi_{\theta }|\psi \rangle$$

Implementing the terminology of quantum computers, we first define a two-dimensional Fock space with states that count photons. Thus, a qubit is identified with the states |1〉 and |0〉 describing the passing or absorption of a photon through the fixed polarizer (the one that determines the final interpretation). These states are associated with the final states |y〉 or |0〉. Following this terminology, we can say that before the final stage of the collapsing interpretation, a photon that travels through the rotated polarizer is in the state,

(17) $$|\phi \rangle =\cos\theta_{\phi }|0\rangle +\sin\theta_{\phi }|1\rangle ,$$

with

(18) $$\cos\theta_{\phi }=\langle 0|\theta_{\phi }\rangle ,\;\;\;\;\sin\theta_{\phi }=\langle 1|\theta_{\phi }\rangle ,$$

where *θϕ*, is measured as the relative angle between the rotated and fixed polarizers.

When the first polarizer is rotated to $45^{o}$ relative to the fixed polarizer, the probabilities of detecting Olive or Yana are equal.

## 5 High dimension extension for interpreting machine

Our proposed device was designed to interpret two alternatives: Anna and Olive. Now we will define a machine capable of analyzing more data.

Our proposed device was designed to interpret two alternatives: Olive and Yana. We now define a machine capable of analyzing more data. Assuming that our device receives only visual information, it consists of several images that form one scenario that is subject to interpretation. To achieve interpretation, we replaced the rotatable polarizer from the previous scenario with several polarizers glued to each other. From a physical perspective, we created an entangled polarized state. Multipolariser rotation was is performed according to the mode calculated by the processor. Another polarizing set was is fixed in place. Similar to a single polarizer, each subpolarizer was is responsible for the local collapse of information from the subpolarizer in front of it. Eventually, after the collapse, the entangled states represented the interpretation. We observed that the presented optical devices indicate the theoretical ability to build machines capable of interpretation. The design of the device based on electronic means appears to contribute significantly to the realization of this construction.

## 6 Relations to AI and ML

If we treat artificial intelligence (AI) and machine learning (ML) as systems that perform measurements in an environment and provide a single interpretation that matches human understanding, uncertainty and ambiguity present a severe problem. We can hypothesize that AI ML machines will report difficulty in determining whether our image is of a young or mature woman. Despite all practical techniques, anticipating ambiguous scenarios [22] that require interpretation, such as classifying an image as Yana or Olive, as demonstrated in this study is sometimes difficult.

The difficulty faced by AI in analyzing ambiguous data raises the question of its expected response in such scenarios. Since the role of this machine is to adapt to human reality, the following question is reasonable: What are the ambiguous scenarios in human society?

Although decoding ambiguity is complex, it can be used as a growth engine to create new and imaginary concepts. Moreover, a combination of clarity and ambiguity contributes to the development of new strategies, such as in organizations. [23]

Therefore, if we exclude the traditional role of AI in providing a single valid interpretation adapted to pre-defined definitions, using interpretation machines to derive new concepts and strategies is possible.

Intelligent systems are often based on ML, where implementing deep learning machines (machines based on artificial neural networks) is preferred over shallow machine learning models and traditional data analysis approaches [24]. Unlike conventional approaches wherein a sophisticated algorithm is responsible for building the model, our system implements a rapid quantum collapse tool [25].

## 7 Summary

Scientific approaches generally search for a single truth to explain phenomena while avoiding various descriptions. Conversely, in human perception (and that of most animals), any observed phenomenon may have several interpretations. In the article, we demonstrated this using ambiguous images that were interpreted as the images of an old or a young woman. Following the current developments in artificial intelligence, the search for an interpretive machine has become more common. In this respect, we hope that this study can advance knowledge toward this direction.

At the beginning of the study, we described the interpretation process in general terms. We then proposed an application for a simple device capable of interpreting example of ambiguous figures. We hope that despite the device's simplicity, this can be a starting point for designing more sophisticated devices.

The most significant difficulty lies in how the device builds a state out of the mixed states received as input. In this article, we needed to convert incoherent photon states arriving from an image into a pure form that would sustain the quantum interpretation process. The solution involved an algorithm to calculate a mathematical set of amplitudes to construct a mathematical representation of a state. Manifesting this set into an actual state corresponds to object rotations (a polarizer). Typically, one may consider a machine that measures incoherent states and establishes a set of amplitudes to represent this input. Associating the collection of these amplitudes with a single object (such as a polarizer) will define the state in which the interpretation process will be performed.

There are computer programs in which an algorithm offers different interpretations for the same event. As all computer activities are deterministic, we cannot consider them as interpretation activities. Our proposed system's collapsed-based interpretation process was entirely random. Thus, without the ability to track the function determining the process's result, there is no way to predict the output. We regarded this as a natural interpretation process. In other words, the proposed collapse output is read only by the observer so that outside observers cannot know the result of the interpretation without communicating with the device. This recognition of the observer role leads us to the familiar “hard problem of consciousness” [26] in the field of philosophy. Reducing all mental phenomena to purely brain biochemical functions without reference to the individual's sensations (interpretations, in our translation) is controversial. Use of machines, even a simple one like ours, to show that the activities of an interpretation machine make sense require an “internal observer.” We suggest the self-observer as the concept with the potential of bridging the biochemical perspectives and the self-interpreting ideas.

When analyzing a device that performs an interpretation (for example, for the images of Yana and Olive), no observer in the device read the results. As suggested in Ref. [27], this defines the difference between a machine; it may be intelligent and a living being. In a living organism, an internal observer reads the interpretation results (consciousness, perhaps).

## CRediT authorship contribution statement

Yehuda Roth: Conceived and designed the experiments; Performed the experiments; Analyzed and interpreted the data; Contributed reagents, materials, analysis tools or data; Wrote the paper.

## Declaration of Competing Interest

The authors declare that they have no known competing financial interests or personal relationships that could have appeared to influence the work reported in this paper.

## Data Availability

No data was used for the research described in the article.
